# Thermal Aggregation of Recombinant Protective Antigen: Aggregate Morphology and Growth Rate

**DOI:** 10.1155/2013/751091

**Published:** 2013-02-13

**Authors:** Daniel J. Belton, Aline F. Miller

**Affiliations:** ^1^Department of Chemical and Biological Sciences, University of Huddersfield, Queensgate, Huddersfield HD1 3DH, UK; ^2^School of Chemical Engineering and Analytical Science and Manchester Interdisciplinary Biocentre, University of Manchester, 131 Princess Street, Manchester M1 7DN, UK

## Abstract

The thermal aggregation of the biopharmaceutical protein recombinant protective antigen (rPA) has been explored, and the associated kinetics and thermodynamic parameters have been extracted using optical and environmental scanning electron microscopies (ESEMs) and ultraviolet light scattering spectroscopy (UV-LSS). Visual observations and turbidity measurements provided an overall picture of the aggregation process, suggesting a two-step mechanism. Microscopy was used to examine the structure of aggregates, revealing an open morphology formed by the clustering of the microscopic aggregate particles. UV-LSS was used and developed to elucidate the growth rate of these particles, which formed in the first stage of the aggregation process. Their growth rate is observed to be high initially, before falling to converge on a final size that correlates with the ESEM data. The results suggest that the particle growth rate is limited by rPA monomer concentration, and by obtaining data over a range of incubation temperatures, an approach was developed to model the aggregation kinetics and extract the rate constants and the temperature dependence of aggregation. In doing so, we quantified the susceptibility of rPA aggregation under different temperature and environmental conditions and moreover demonstrated a novel use of UV spectrometry to monitor the particle aggregation quantitatively, *in situ*, in a nondestructive and time-resolved manner.

## 1. Introduction

The study of protein aggregation is a burgeoning field of research driven by the urgent need to elucidate the mechanism of neurodegenerative diseases, the desire to understand and mimic natures' ability to create hierarchical complex nanostructures, and the necessity to understand and minimise product loss during the processing and formulation of biopharmaceuticals. Aggregation is of particular importance for therapeutic proteins as it can lead to a loss of product, reduce efficacy, alter biological activity and pharmacokinetics, and even raise safety concerns such as increased immunogenicity [1–3]. Aggregation can be induced by solution conditions such as protein concentration, pH, salinity, temperature, and the presence of additives [4, 5]. These variables are also known to affect the quantity and morphology of aggregate formed [5]. Stresses to the protein such as over-expression, refolding, freeze-thaw cycles, agitation, or exposure to hydrophobic surfaces or air (including foaming) can also lead to the formation of aggregates [2, 4–6]. Each of these environmental factors is typically encountered during bioprocessing, downstream processing, storage, and also during and after *in vivo* delivery of biopharmaceutical actives. Hence, there is significant on going work channelled into exploring the onset of aggregation and the aggregation pathway. Understanding these factors subsequently allows the development of an informed strategy to minimise aggregation during biopharmaceutical production, for example, the inclusion of surfactants to influence protein monomer interactions.

The influence of solution pH on aggregation is one of the more studied and important parameters that controls the onset of aggregation and final aggregate morphology [5]. This is because pH alters the surface charge of the protein monomer and also the extent of any structural disruption prior to aggregation, and hence influences their propensity to self-assemble and the manner in which they go onto aggregate. For example, at pH values close to the isoelectric point, repulsive interactions between native monomers are reduced, making assembly more favourable. Under these conditions, particulates typically form [7]. At pHs far from the isoelectric point, increased charge repulsion within the protein destabilises the folded conformation, leading to the exposure of hydrophobic groups, which in turn can drive the self-assembly of these unfolded states, typically into *β*-sheet rich fibrillar structures [8, 9]. Temperature is another key factor, if not the most critical factor in commercial processes that induces aggregation, where increasing the temperature increases the vibrational motion and diffusion of proteins which is a necessary step for aggregation. Moreover, as the temperature nears the denaturation temperature of the protein, the protein partially unfolds, exposing hydrophobic regions, which induces aggregation [10].

The literature is awash with many studies that postulate different models for the self-assembly of proteins, and these can generally be divided into two main categories: empirical or mechanistic [11]. Mechanistic models are based on a reaction scheme and have parameters relating to the kinetics/thermodynamics of the process, whereas empirical models utilise functions that fit the data but have no physical meaning. A large array of different mechanistic models have been proposed, and these have been broadly categorised as monomer addition, reversible association, prion aggregation, minimalistic 2-step, or quantitative structure-activity relationships [11]. Alternatively, Roberts proposed a comprehensive generic mechanistic scheme for protein aggregation with associated generic equations, and he went onto show how these could be simplified for certain conditions and limiting cases [12]. Overall, most of these models are based on the idea of aggregation being mediated by a reactive intermediate which is in equilibrium with the native state; the intermediate is able to aggregate, initially via a nucleation step followed by a growth phase, that is, Native (N) ↔ Intermediate (I) → Aggregate (A). Many groups have experimentally tested such models, and techniques have been developed and exploited to study aggregation both *ex situ* and *in situ* [13, 14]. In *ex situ*, the state of aggregation is measured in static samples, where long data acquisition times are needed, or samples need to be separated, dried, fixed or labelled, for example, using electron microscopy or mass spectrometry. Furthermore, the aggregation process has to be reliably halted and persevered for a series of samples representing different stages. In *in situ* measurements, aggregation events are monitored as they happen, but the technique used needs sufficient time resolution for the process being monitored and the data analysis can be complex, since there is typically a mixture of component sizes, that is, aggregates and monomers. Typical techniques currently used include circular dichroism, Fourier transform infra-red, and dynamic light scattering [13, 15]. For a technique to yield useful information about the aggregation process, it is crucial to be able to relate the measured parameter back to changes in the aggregation state such as aggregate size or monomer depletion.

Here, the thermal aggregation of an industrially relevant biopharmaceutical recombinant protective antigen (rPA) (active component in a second-generation anthrax vaccine [16]) has been examined visually and via turbidity measurements, before information on the size and the shape of the aggregates formed were obtained using a combination of optical and environmental scanning electron microscopies. Two environmental conditions were explored, where samples were prepared with and without the denaturant urea. Subsequently, the rate of aggregate growth at different isothermal temperatures was compared using ultraviolet light scattering spectroscopy (UV-LSS) [17–23], with particle size being extracted as a function of time. This was done by collecting spectra over time without the need to remove material for analysis, hence demonstrating a nondestructive, *in situ,* and time-resolved method for monitoring the aggregation process. Results for the aggregation of rPA will be discussed, and a model describing the kinetics and thermodynamics of aggregation is presented.

## 2. Materials and Methods

### 2.1. Materials

rPA was supplied by Avecia Biologics (UK) at 2 mg mL^−1^ in a phosphate-buffered saline solution adjusted to pH 7.4. Doubly distilled water was obtained from an Elga PureLab Ultra (18.2 Ω). All other chemicals were purchased from either Sigma-Aldrich (UK) or Acros Organics (UK), where the reagent grade is at least 97% pure and used as recieved. 

### 2.2. Sample Preparation

The rPA samples supplied were treated using the following procedure to provide a consistent starting material for analysis. Initially, rPA was precipitated from solution by heating (50°C for circa 5 min). The resulting gel was centrifuged at 6000 rpm for 1 minute, and the supernatant was discarded. The gel was resuspended by adding doubly distilled water and vortexing for 2 min. The sample was centrifuged as before, and the supernatant was discarded. The gel was resolubilized by adding urea and doubly distilled water to form an 8 M urea solution. rPA was refolded by dilution, using 1 part rPA in 8 M urea to 31 parts refold buffer. This was done in two stages, with a 1 : 7 dilution followed by 1 : 4 dilution 2 minutes later. The refold buffer contained 25 mM TRIS, 25 mM NaCl, 2 mM CaCl_2_ and was adjusted to pH 7.4 with hydrochloric acid. The final buffered samples contained between circa 0.15 and 0.3 mg mL^−1^ rPA with 0.25 M urea and were analysed immediately after refolding. This concentration was chosen as it mimics the conditions of storage of some of the formulations of protective antigen vaccines. Samples without urea were prepared using the refolding method followed by dialysis using 3500 Dalton molecular weight cutoff Visking dialysis membrane (Medicell International Ltd) against 10 times excess of chilled refold buffer for 18 hours in the refrigerator (~4°C). After this, the external solution was replaced twice with fresh refold buffer and allowed to dialyse for a further 24 hours. The rPA sample was then recovered from the sealed membrane and stored in the refrigerator (circa 4°C) prior to use. This was done to reduce any degradation of the rPA during storage. Samples, however, were not frozen to avoid potential freeze-thaw damage.

### 2.3. Concentration Analysis

The concentration of the refolded rPA solution was determined from its UV absorbance at 280 nm (Shimadzu UV 2501-PC spectrophotometer), using a molar absorption coefficient of 72769 M^−1^ cm^−1^.

### 2.4. Visual Observations

The isothermal aggregation of rPA was monitored visually over time by incubating samples over a range of temperatures (43–49°C) close to the denaturation temperature of rPA (50.0°C) by using a recirculating water bath connected to a heating stage that contained the sample cell. The temperature was measured using a calibrated K-type thermocouple placed in the sample solution (accurate to ± 0.3°C). 

### 2.5. Optical Microscopy

Images of rPA aggregates were obtained using a Zeiss Axioplan 2 in transmission mode with a 10x magnification objective and a digital camera. The aggregated samples were pipetted onto a microscope slide and a cover slip placed on top.

### 2.6. Environmental Scanning Electron Microscopy (ESEM)

Aggregates were mounted for ESEM analysis simply by lifting them out of solution on a mica disc and placing them directly onto the sample stage. The samples were examined using a Philips FEI Quanta 200 ESEM with the electron gun accelerating voltage set to 30 kV, the sample stage at 5°C, and the chamber pressure at 6 torr.

### 2.7. Light Scattering

Light scattering spectra were recorded using a Shimadzu UV 2501-PC spectrophotometer set to record between 250 and 390 nm every 2 nm at a medium scan rate. The sample was heated *in-situ* as previously described. 

### 2.8. Theory

The wavelength dependence of absorbance arising from light scattering by particles in solution follows the relationship [24]

(1) $$\begin{array}{c} A_{\lambda }=\alpha \lambda^{-\beta }, \end{array}$$

where *A*_*λ*_ is the absorbance at wavelength *λ*, *α* is a constant and *β* is the scattering exponent. The scattering exponent can be related to particle size using Mie theory. Here, the Mie equations were solved over a range of particle sizes (nanometres to micrometres) for wavelengths between 320 and 390 nm using a FORTRAN programme adapted from Bohren and Huffman [24]. These calculations required the refractive index of the particles and the surrounding solution for all relevant wavelengths. The solution refractive index was taken to be that of water [25] and the particle refractive index was calculated by evaluating the Lorentz-Lorenz molar refraction [26] using the chemical formula of rPA and a density of 1.43 g cm^−3^. The density was based on the molecular weight of rPA (82667 Da) and its volume (95.74 nm^3^) as calculated by VADAR [27] using the crystal structure of rPA [28], which is stored in the protein data bank (http://www.pdb.org/) [29] under PDB ID : 1ACC. This value of density was considered reasonable compared to the density of other proteins with a similar molecular weight [30]. The values of refractive index, *n*, calculated for the particles at different wavelengths, *λ*, within the visible region are given in Table 1. These values were subsequently extrapolated to the UV region using the Cauchy equation [26];

(2) n=1.642+7980λ2.

The results obtained using Mie theory were analysed to give a theoretical scattering exponent versus diameter, providing a means of converting experimental scattering exponents to particle size:

(3) d=−0.686 β6+6.869 β5−21.397 β4+23.795 β3−23.682 β2−111.896 β+642.207,

where *d* is the particle diameter (nm) and *β* is the scattering exponent between 320 and 390 nm.

## 3. Results and Analysis

### 3.1. Qualitative Aggregation

**Figure 1:**
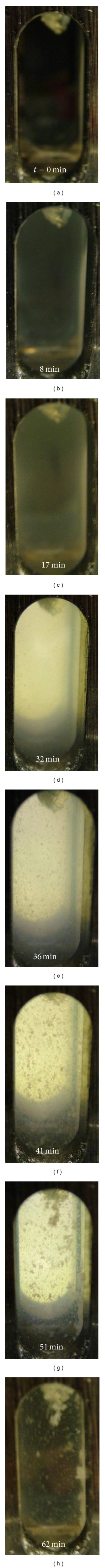
Photographs taken at different time points of rPA aggregates forming in solution at 47.6°C. Note that each of (a) to (c) and (h) has a black background placed behind the cuvette and (d) to (g) have no background allowing light to enter the sample from behind.

 Solutions of rPA (0.31 mg mL^−1^) were incubated over a range of temperatures (25–50°C), and their visual appearance was monitored over time. This temperature range was selected as the denaturation temperature of rPA is known to be ~50°C [31]. Sample appearance was observed to change when the temperature increased above ~43°C. A typical example of these changes is recorded in Figure 1, which shows the visual appearance of rPA in solution over time whilst held at a steady temperature of 47.6°C. Initially, the sample was clear, as can be seen from Figure 1(a), and remained clear over the first few minutes. After 8 minutes, the sample started to become cloudy, which is evident in Figure 1(b). The slightly cloudy homogeneous appearance of the sample indicates the presence of microscopic particles of sufficient size and concentration to noticeably scatter visible light. Such behaviour is indicative of the aggregation of rPA. Over the minutes that followed, the sample remained homogeneous but gradually became more turbid, suggesting further aggregation. This can be seen by comparing the images in Figures 1(b) and 1(c); the latter appears cloudier. After 32 minutes, the sample was no longer homogeneous, since particles large enough to be observed visually started to form (see Figure 1(e)). From this point forwards, the black background was removed to provide improved contrast for observing the macroscopic particles that were forming in the solution; the sample remained cloudy but appeared brighter, since light was able to enter from behind. The macroscopic particles were more easily observed and appeared as dark spots against the bright cloudy solution. Following their emergence, the macroscopic particles were observed initially to increase in size and number as can be seen from the images shown in Figures 1(e) and 1(f). Beyond this point, the number of individual particles appeared to fall whilst continuing to grow in size (Figure 1(g)). This observation suggests that the particles grew by clustering together. The final image of the sample, Figure 1(h), was taken with the black background replaced to enable comparison with the initial sample appearance. It shows that the sample had returned to a clear solution apart from the presence of large white particles, some of which had settled on the bottom and the sides of the cuvette. Such observations suggest that small microscopic particles form initially, causing the sample to appear turbid, and subsequently cluster to form large macroscopic particles that sediment due to gravity when they are above a critical size.

**Figure 2:**
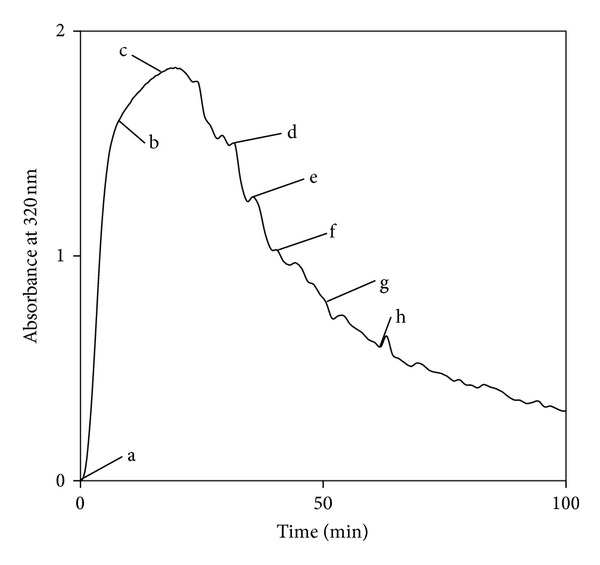
Absorbance at 320 nm versus time for rPA held at 47.7°C (×). Labels “a” to “h” relate to the photographs shown in Figure 1.

 These visual results were complemented by turbidity measurements recorded under identical conditions (see Figure 2); the labels (“a” to “h”) in Figure 2 are positioned to relate to the photographs taken of rPA aggregation shown in Figure 1. The turbidity was assessed by recording the optical density using light with a wavelength of 320 nm. Figure 2 shows a sharp rise in turbidity over the first 20 minutes of incubation. This is consistent with the appearance and growth of particles in the solution, as indicated by the visual observations. The subsequent fall in turbidity suggests that the particles were either decreasing in size (which might occur if the aggregation was reversible) or decreasing in concentration (which could occur if the particles clustered to form larger structures). The latter is consistent with our visual observation of particles growing in size and reducing in number over time, before settling to the bottom of the sample cuvette.

 Similar visual and turbidity observations were noted for other samples incubated between 43° and 49°C, as all appeared to have a similar two-step mechanism. The time scales for each step varied widely; however, for the different temperatures, the time to reach peak turbidity ranged from circa 10 minutes at 49°C to 10 hours at 43°C. This dramatic increase in aggregation rate versus temperature could be driven by either increasing the translational kinetic energy of protein monomers, causing more frequent collisions between the particles, or increasing the internal kinetic energy of the protein monomers. The latter will drive monomers toward an unfolded state, with the associated exposure of hydrophobic patches increasing the likelihood that a collision results in self-association.

### 3.2. Aggregate Morphology

**Figure 3:**
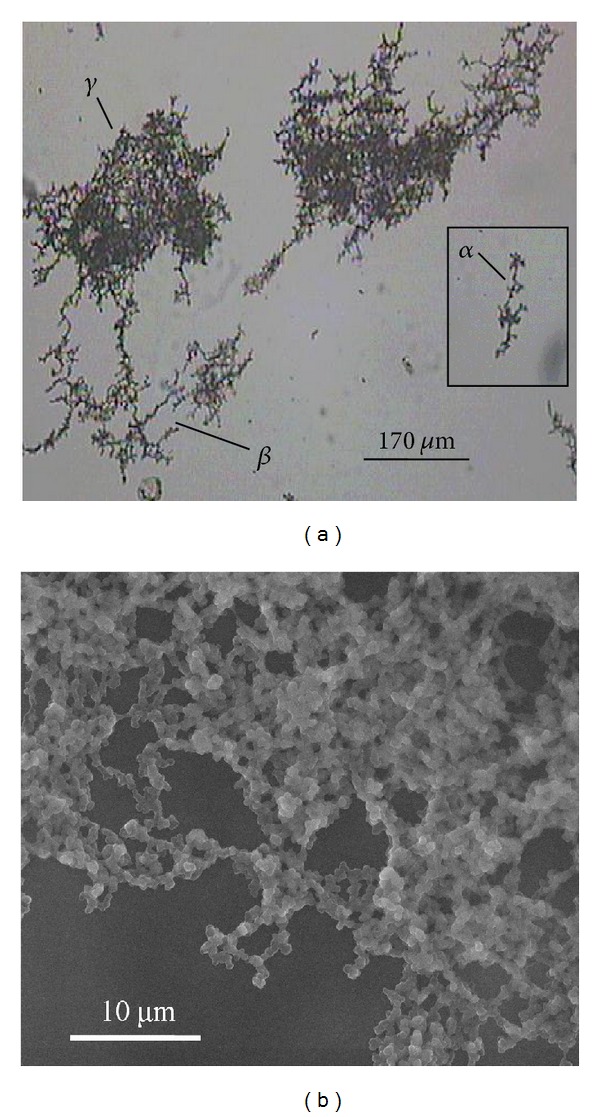
rPA aggregates formed after incubation at 47°C for 16 hours: (a) optical micrograph, 10x magnification (170 *μ*m scale bar), and (b) ESEM micrograph taken with a sample temperature of 5°C, 6 torr chamber pressure, and 30 kV electron gun accelerating voltage (10 *μ*m scale bar).

The morphology of the macroscopic particles formed after incubation was examined using optical and electron microscopy.  Figure 3(a) shows a typical optical micrograph of the rPA aggregates formed after 16 hours at 47°C. The micrograph reveals the formation of several aggregates with different shapes and sizes, but all appear to be composed of microscopic particles. This is consistent with the visual observation of a cloudy solution of microscopic particles which cluster to form large aggregates. The aggregates generally range in length from ~75 to 730 *μ*m and have a width of ~5–150 *μ*m. Three aggregates with fairly distinct structures have been labelled *α*, *β*, and *γ* in Figure 3(a). The smallest, labelled *α*, is 165 *μ*m long and 30 *μ*m at its widest point. This aggregate appears reasonably linear with growth predominantly in one direction with limited side branching. The other two aggregates, *β* and *γ*, are larger and have similar dimensions: ~380 *μ*m by 150 *μ*m. However, *γ* has a very dense structure, whilst *β* has a more open structure. The open structure of *β* consists of a branched system of linear components similar to aggregate *α*, suggesting that *α* is a precursor to the formation of *β*: either as one of many components that come together or as the starting point for further growth or a combination of both. The structure of *γ* is densely packed; however, there is still some evidence of a branched structure and these are similar to the structural features observed in *β*. Suggesting that *β* is possibly a precursor to the formation of *γ*. Such observations and inferences are also supported by previous work on fractal aggregates, reviewed by Meakin [32]. Such comparisons suggest that the rPA aggregates described here are fractal aggregates, confirming that they are formed by the clustering of microscopic particles. To obtain further information on the size and the shape of the microscopic particles, the aggregates were viewed under ESEM and a typical micrograph is given in Figure 3(b). The microscopic particles appeared reasonably spherical and uniform with a diameter of approximately 500 nm. This observation is consistent with the previously reported results for the thermal aggregation of proteins close to their isoelectric points, which showed the formation of monodisperse particulates [7]. Here, we are working at pH 7.4, which is close to the isoelectric point of rPA (pH 5.6) [16]; therefore, individual protein monomers will have reduced net charge. In addition, the salt present in the buffered media will screen any remaining charge on the proteins, thus any long-range charge-charge repulsions which could act as a barrier to aggregation will have been minimised. The protein monomer is likely to be partially unfolded as the temperature approaches the denaturation temperature (~50°C). The exposed hydrophobic regions will consequently drive nonspecific monomer aggregation under these conditions of reduced net charge. This in turn leads to the formation of the three-dimensional spherical aggregates, as observed in Figure 3(b). The approximately uniform size of the particles in each sample suggests that their concentration remained reasonably constant during their growth which is consistent with the features of protein aggregation summarised by Gosal and Ross-Murphy [10]. A slight increase in particle diameter from 360 to 500 nm was noted as the incubation temperature increased from 43 to 49°C. 

### 3.3. Particle Growth Rate

**Figure 4:**
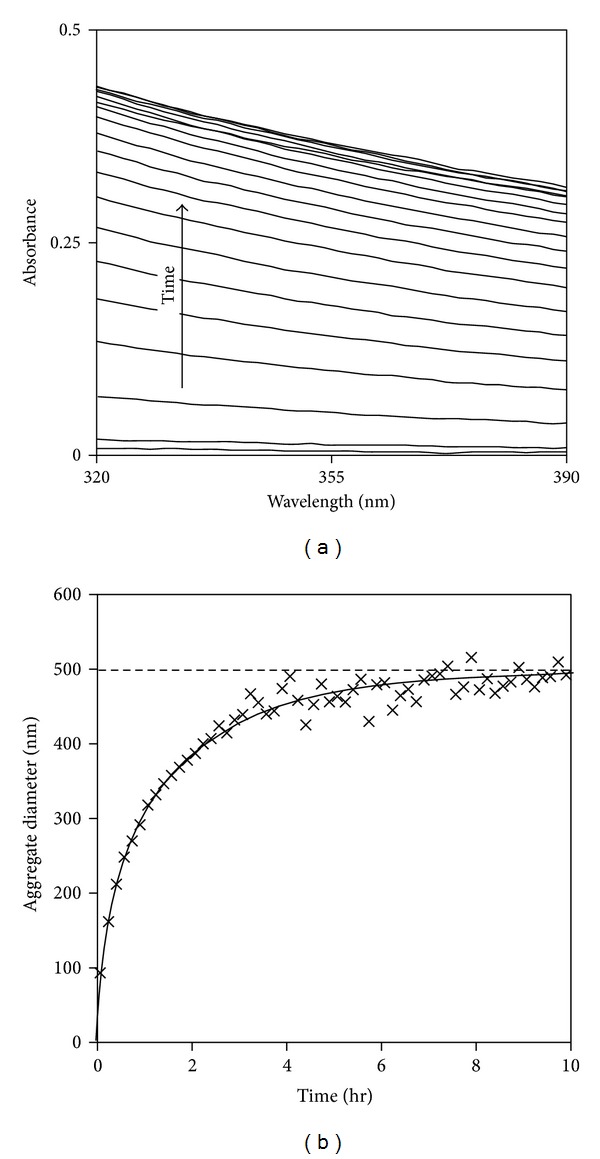
(a) Change in UV light scattering arising from the growth of rPA aggregates at 45°C, spectra collected in 10-minute intervals for 0–200 minutes, and (b) aggregate diameters calculated from light scattering spectra as a function of time.

The rate of aggregation at different isothermal temperatures was explored using UV light scattering spectroscopy (UV-LSS), where spectra were recorded every 20 seconds initially and then every 20 seconds to 40 minutes depending on the aggregation rate. Such fast acquisition times provide a speed advantage over other light scattering techniques for analysing such aggregation kinetics. Typical results for incubation at 45°C are shown in Figure 4(a). It is clear that there was no absorbance initially (no chromophores in the protein absorb over this wavelength range), but the absorbance intensity increases over time, where the difference between successive spectra is large initially but reduces over longer times. Minimal difference was observed after ~200 minutes. This implies that the aggregates grew quickly initially when the rPA monomer concentration was at its highest, followed by slowing growth as the rPA monomers were consumed and falling in concentration. This suggests that the particle growth rate is limited by rPA monomer concentration. 

As discussed previously, rPA solutions became turbid during incubation at temperatures ≥43°C; therefore, it can be assumed that multiple light scattering was occurring. It can be assumed, however, that the wavelength dependence of light scattering obtained by solving the Mie equations would hold for the scattering occurring here. As such, the UV spectra in Figure 4(a) were analysed (see Section 2.8) to give aggregate diameter versus time; see Figure 4(b). It is clear that the particles grew quickly over the initial ~60 min before gradually slowing and reaching a final particle size after ~300 min at this temperature. The equilibrium sizes of particles formed increased slightly from 390 to 500 nm with increasing the incubation temperature, which correlates well with the ESEM observations (~360–500 nm). This confirms our assumption that the wavelength dependence of light scattering in a turbid solution can be adequately approximated by Mie theory.

**Figure 5:**
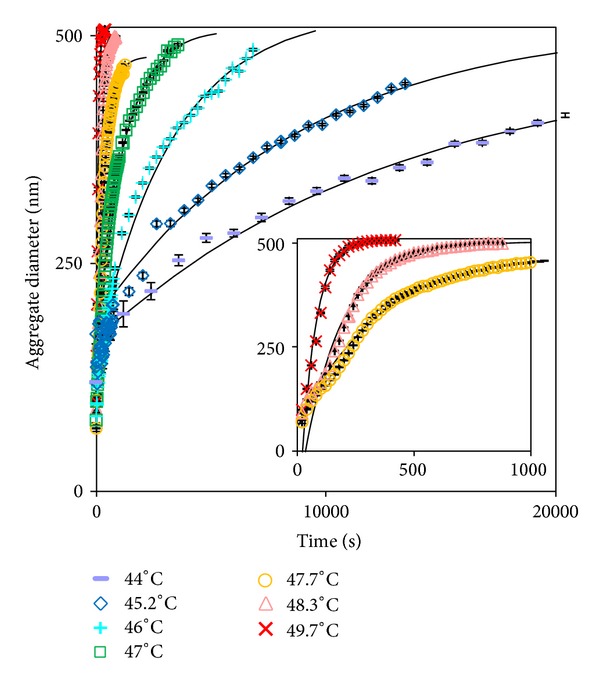
Comparison of aggregate diameter calculated from light scattering spectra versus time for rPA samples with 0.25 M urea held isothermally at various temperatures (the inset highlights higher temperature runs).

To explore the effect of isothermal temperature on particle growth rate and consequently gain an insight into the aggregation kinetics, particle size was recorded as a function of time for a range of temperatures (43°–49°C) and the results are given in Figure 5. The aggregate diameter versus time profile was fitted with

(4) $$\begin{array}{c} d_{\text{model}}=d_{f}-d_{0}\cdot e^{-t/\tau }, \end{array}$$

where *d*_model_ is the model fit to the particle diameter (nm), *d*_*f*_ is the final particle diameter that the function converges on (nm), *d*_0_ is a fitting parameter (nm), *τ* is a time constant for the particle growth process (s), and *t* is time (s). The optimum fit was found using Newton's method to maximise the correlation coefficient, *R* ^2^, whilst allowing the value of *d*_*f*_ to vary. The values of *d*_0_, *τ*, and correlation coefficient were calculated from a straight line fit (using least squares regression) to a plot of ln⁡(*d*_*f*_ − *d*_model_) versus time. The model fit for each run is included in Figure 5.

**Figure 6:**
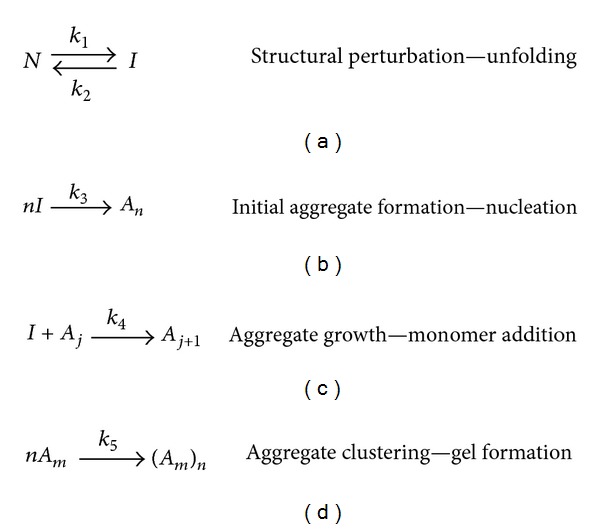
Schematic representing the 4 possible steps in the proposed kinetic model of rPA aggregation: (a) structural perturbation, (b) initial aggregate formation, (c) aggregate growth, and (d) aggregate clustering.

All profiles show an increase in diameter over time; however, the time taken to reach the maximum diameter is markedly different for each temperature: the higher the temperature, the shorter the time scale to reach the maximum diameter. This is reflected in the results by a notable increase in the profile gradient when the temperature is increased, where the maximum gradient increases by 2.7 times on average for every 1°C increase in temperature. Similar results were obtained for a range of samples containing an additive: 0.25 M urea. In this case, similar trends were observed over time for a range of incubation temperatures, and the only difference arising was in the rate of particle growth being slightly faster for each sample in the presence of 0.25 M urea. All samples contained a homogeneous distribution of particle size (confirmed by ESEM) suggesting that the aggregate concentration remained constant over time. This means that the quantity of rPA in the aggregates can be determined by estimating the aggregate concentration and using the ratio of aggregate to monomer volume. From this, the difference between the quantity of rPA incorporated within aggregates and the quantity of monomer present initially gave monomer concentration over time, providing a possible means of assessing the aggregation kinetics. To this end, data over initial incubation times (where the scattering was increasing) were analysed using a generalised scheme of the protein aggregation pathway. This scheme is outlined in Figure 6, where *N* is the native state, *I* is the protein monomer in an intermediate (unfolded or denatured) conformational state, *A*_*j*_ is an aggregate consisting of *j* protein molecules, (*A*_*m*_)_*n*_ is a cluster of particulate aggregates, and *k*_1_ to *k*_5_ are the rate constants for the different processes. This aggregation pathway can be simplified based on the aforementioned experimental observations; it was considered reasonable to exclude *step d,* since the spherical nature of the aggregates inferred that their growth was dominated by single monomer addition rather than clustering to form irregular structures, and *step b* was also considered not to have played a significant role, since the aggregates formed were reasonably uniform in size, for which the aggregate concentration would have had to be reasonably constant during their growth. It is reasonable to expect that if new aggregates had formed throughout the aggregation process, then a wide distribution of aggregate sizes would have been observed. The modelling of the aggregation kinetics was further simplified by considering two limiting cases: unfolding limited aggregation and association-limited aggregation. Both were tried and association-limited aggregation was found to be the most appropriate as it gave a better fit to the experimental data and more reasonable kinetic values in comparison to previous work [33, 34].

In the case of association-limited aggregation, *step a* is more rapid than *step c,* (*k*_1_ and *k*_2_⋙*k*_4_). As such, *N* and *I* come to pseudoequilibrium, hence *k*_1_ *C*_*N*_ ≈ *k*_2_ *C*_*I*_. The total monomer concentration, *C*_*M*_, is the sum of the concentrations of the monomers in the native state (*N*) and the structurally altered state (*I*), that is, *C*_*M*_ = *C*_*N*_ + *C*_*I*_. Combining these two relationships gives

(5) $$\begin{array}{c} C_{M}=\frac{\left(1+K\right)C_{I}}{K}, \end{array}$$

where *K* is the equilibrium constant, *K* = *k*_1_/*k*_2_. Since *step c* is the rate limiting step, the resulting kinetic model is a second-order rate equation, which incorporates the equilibrium constant in order to be stated in terms of *C*_*M*_,

(6) (−rM)=k4CMCAK(1+K).

As stated previously, the aggregate concentration, *C*_*A*_, is expected to have been reasonably constant; therefore, the model can be reduced to pseudo-first order (7), where the equilibrium constant is incorporated into the rate constant for the rate equation (8) as follows:

(7) (−rM)=kpseudo CM,

(8) kpseudo=k4CAK(1+K).

This model was subsequently used to estimate the aggregation kinetics of all samples using the experimental particle diameter, *d*(*t*), (Figure 5) to calculate the number of monomers in each aggregate as a function of time (*A*_*N*_(*t*)) from the ratio of the monomer volume to aggregate volume via

(9) AN(t)=π[d(t)]36VM.

The volume of a PA molecule (*V*_*M*_) was taken to be 95.74 nm^3^, which was obtained using the crystal structure, as described previouslt. The free monomer concentration versus time (*C*_*M*_(*t*)) was obtained by taking the difference between the initial monomer concentration (*C*_*M*0_) and the amount of monomers in the aggregates, based on the previous assertion that the aggregate concentration remained constant,

(10) CM(t)=CM0−[CA×AN(t)].

The final size of aggregate that the function converges on is assumed to be the size where aggregates would have stopped growing if the clustering process is not interfered with the particulate growth phase. As such, this is the point at which all the protein monomers would have been consumed and all the proteins would have been present in the form of the particulate aggregates. The aggregate concentration is therefore given by;

(11) CA=CM0 6VMπ  (df)3.

**Figure 7:**
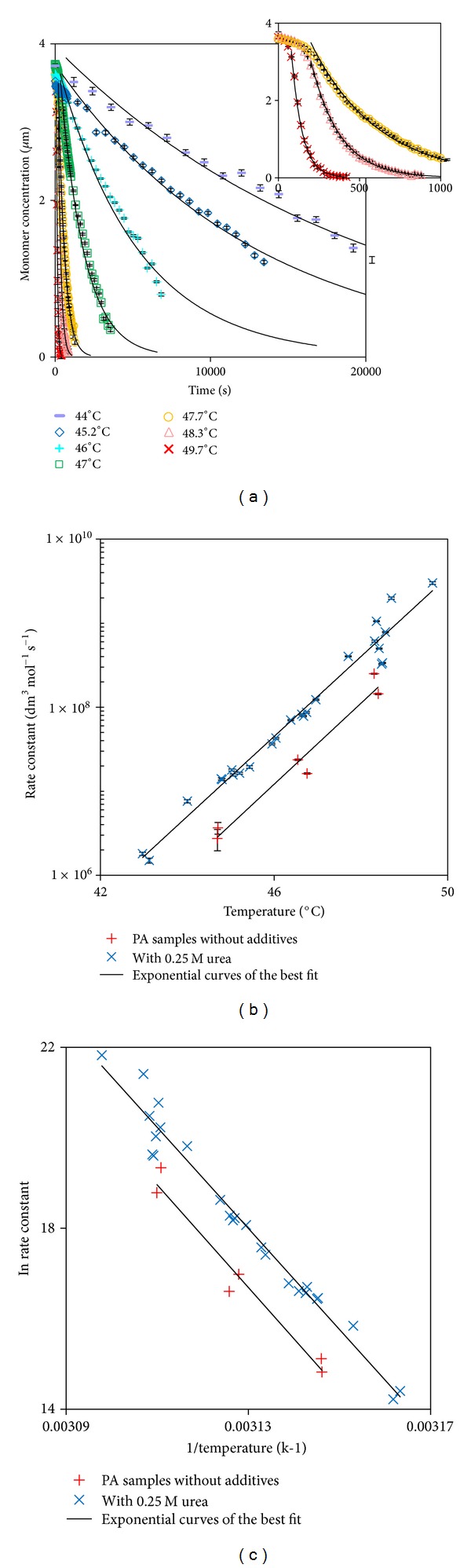
(a) Monomer concentration as a function of time for rPA samples with 0.25 M urea held isothermally at various temperatures (the inset highlights higher temperature runs). (b) Second-order association-limited aggregation rate constant versus temperature. (c) Arrhenius plot.

This procedure was used to generate concentration versus time profiles for the best fit pseudo first order kinetics over a range of temperatures, and the results for the 0.25 M urea samples are shown in Figure 7(a), and the values for the second-order *k*'s are provided in Figure 7(b). It is evident that in each case the fitted data are in good agreement with those obtained experimentally and the magnitudes of the rate constants obtained from these data are reasonable, although a little high, compared to those reported elsewhere for the association-limited aggregation of protein (e.g., bovine granulocyte colony-stimulating factor) [33]; extracted values were in the region of 10^4^ to 10^6^ dm^3^ mol^−1^ s^−1^ over a similar temperature range. It is clear from Figure 7(b) that the association-limited aggregation rate constants for samples with and without added urea increase exponentially with temperature. On average the rate constant increases three-fold for every 1°C incre ase. If the data are re-plotted as ln⁡*k* versus 1/*T* (Figure 7(c)), it is clear that the data follows Arrhenius' Law,  *k* = *A*_*f*_exp⁡(−*E*_act_/*RT*), where *A*_*f*_ is the pre-exponential factor, *T* is the absolute temperature (*K*), *E*_act_ is the activation of the reaction (J mol^−1^) and *R* is the ideal gas constant (J mol^−1^ K^−1^). From the gradients of each slope the activation energies for the two sample types were calculated to be 942.0 ± 92.4 kJ mol^−1^ for rPA without additives and 928.8 ± 32.5 kJ mol^−1^ for rPA with 0.25 M urea. These values suggest that the addition of urea reduced the activation energy slightly, which correlates with the observation that the presence of urea increases the rate of aggregation. The magnitude of these activation energies are reasonable compared to those reported elsewhere for the association limited aggregation of protein [12]; reported as being between ~420 and ~840 kJ mol^−1^. These values are reported as “observed” activation energies and are accompanied by the suggestion that the temperature dependence of association limited rate constants does not follow true Arrhenius behaviour [12]. The frequency factors, *A*_*f*_, for the two sample types were calculated to be 1.8 × 10^161^ dm^3^ mol^−1^ s^−1^ for rPA without additives and 4.7 × 10^158^ dm^3^ mol^−1^ s^−1^ for rPA with 0.25 M urea. These values are very high and unlikely to have any physical significance. Published values of the second-order frequency factors for various small molecule solution phase reactions are in the range of 10^2^ to 10^16^ dm^3^ mol^−1^ s^−1^ [35]. This suggests that the Arrhenius equation is no more than an empirical fit to the data here. This is not unexpected given the suggestion cited previously that the temperature dependence of association-limited rate constants does not follow true Arrhenius behaviour. However, the correlation coefficients between the data and the lines of the best fit show a reasonable fit: 0.963 for the samples without additives and 0.973 for the samples with 0.25 M urea. 

An alternative and more appropriate approach to modelling the temperature dependence of the rate constant is to factor in the behaviour of the equilibrium constant, *K*.The analysis using the association-limited model has so far yielded an “observed” second-order rate constant for the aggregation process. The kinetic model for this analysis is represented by

(12) $$\begin{array}{c} \left(-r_{M}\right)=k_{\text{obs}}\;C_{M}\;C_{A}, \end{array}$$

where *k*_obs_ is the observed rate constant. Comparing this with (6) reveals how *k*_obs_ is related to the actual rate constant (*k*_4_) and the equilibrium constant *K*,

(13) kobs=k4K(1+K).

The temperature dependence of *K* is given by [35]

(14) ln⁡(K)=ΔSR−ΔHRT,

where Δ*S* is the entropy change (J mol^−1^ K^−1^), Δ*H* is the enthalpy change for the process (J mol^−1^), *T* is the absolute temperature (K), and *R* is the gas constant (J mol^−1^ K^−1^). To fit the experimental data to the temperature dependence of the equilibrium constant, it was assumed that the rate constant, *k*_4_, is independent of temperature. The physical significance of this is that the activation energy is assumed to be negligible for the process of perturbed monomers being added to the growing aggregate. This is a reasonable assumption, since the activation energies for the reaction of highly reactive free radicals can be close to zero [35, 36], and given that the perturbed monomers will have highly unfavourable hydrophobic patches exposed to water, they too are likely to be highly reactive and easily associated with an aggregate in order to reduce their free energy. Rearranging (13) and equating it to (14) yields

(15) ln⁡(kobsk4−kobs)=ΔSR−ΔHRT.

**Figure 8:**
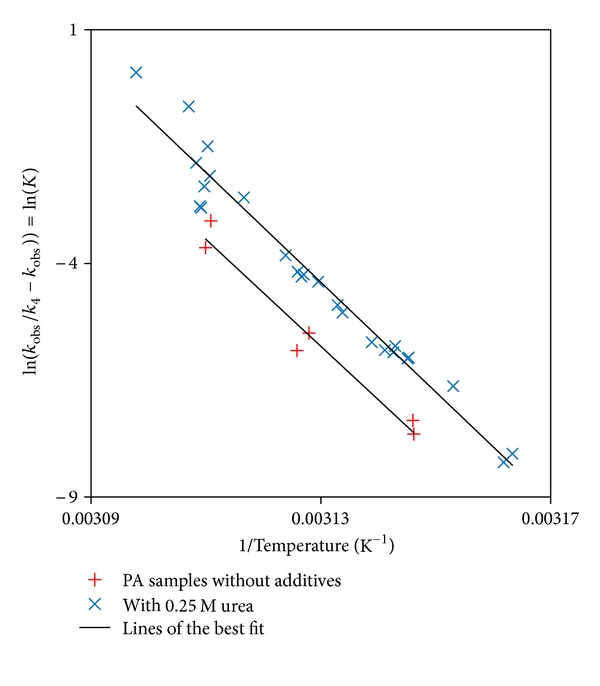
Natural log of equilibrium constant versus the reciprocal of absolute temperature for the association-limited aggregation model.

Therefore, plotting ln⁡(*k*_obs_/(*k*_4_ − *k*_obs_)) versus 1/*T* will yield a straight line, the gradient of which will be −Δ*H*/*R* and the intercept Δ*S*/*R*. The value of *k*_4_ was found by searching for the best fit. The initial estimate for *k*_4_ was picked by choosing a value greater than the largest value of *k*_obs_ so that the logarithmic term could be satisfied. When the 0.25 M urea data was plotted, it was found that the correlation coefficient for the fit between the line of the best fit and the data points improved as the value of *k*_4_ was increased. Eventually, there was no change in the correlation coefficient as *k*_4_ was increased. It was on this basis that an optimum value of 5.77 × 10^9^ dm^3^ mol^−1^ s^−1^ was selected for *k*_4_. The value of *k*_4_ effectively represents the value of the frequency factor, since the activation is assumed to be zero. As such, this optimum value of rate constant/frequency factor is much more likely to have a physical significance, since it is comparable to general values of the frequency factor found in the literature [35] discussed previously. The same value of *k*_4_ was used for the analysis of both sets of data: rPA without additives and rPA with 0.25 M urea. This was done on the assumption that the collision rate would not be significantly altered in the presence or the absence of urea. A plot of the resulting data is shown in Figure 8. It is clear that the correlation coefficients between the data and the lines of the best fit are in reasonable agreement for both sets of samples. Δ*H* and Δ*S* were extracted from the graph for each sample and used to calculate the Gibbs free energy change, Δ*G*, at 25°C. The values obtained (Table 2) were compared reasonably well with those reported in the literature for the Δ*G* between folded and unfolded proteins, reported to typically be between 20 and 60 kJ mol^−1^ [37]. These values also indicate, as expected, that the presence of urea reduces the stability of rPA, most likely by helping to disrupt the native structure of the protein [4], causing it to become perturbed at temperatures lower than those in the absence of any urea. 

**Figure 9:**
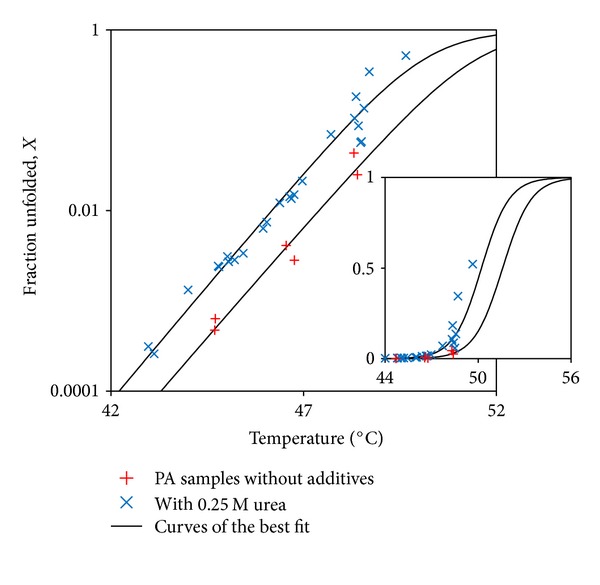
Fraction of monomer in the perturbed state as a function of temperature for the association-limited aggregation model. Curves of the best fit follow (14) and (16) with ΔH and ΔS values from Table 2 (black curve) on logarithmic scale (the inset shows extrapolation to higher temperatures on normal scale).

To explore the effect of temperature on the extent of disruption of individual proteins, the fraction of protein in the perturbed intermediate state, *X*, was calculated using (16) and plotted as a function of temperature (Figure 9):

(16) X=CICM=K(1+K).

Extrapolation of this data to ambient temperatures shows that the fraction of protein in the perturbed state is negligible at low temperatures. For example, extrapolation to 25°C for the sample with 0.25 M urea shows that the fraction of protein in the perturbed state is 10^−14^. This corroborates our experimental observations that rPA does not show observable aggregation over extended periods (circa 16 hours) at ambient temperature. The unfolded fraction has also been extrapolated to higher temperatures, using (11) and (12), and the results have been shown by the extrapolated line of the best fit in Figure 9. The inset of Figure 9 shows that the fraction of monomer in the perturbed state rises sigmoidally between 46°C and 55°C and that a higher proportion of protein is perturbed when urea is present. Both observations correlate with the susceptibility of the protein samples to aggregate, that is, the more protein perturbed, the higher the chances and the faster the kinetics of aggregation. It also links in well with other studies on the thermal unfolding of rPA. For example, 8-anilino-1-naphthalene sulfonate (ANS) and rPA reported elsewhere show that the hydrophobicity of rPA increases sigmoidally versus the temperature between 40° and 55°C [38], as was also the case when the thermal unfolding was examined by circular dichroism, which revealed a sigmoidal increase in unfolding versus the temperature between 45° and 55°C [31].

## 4. Conclusions

The susceptibility of a biopharmaceutical protein rPA to aggregate as a function of temperature and formulation conditions has been determined, and the kinetics and thermodynamics of the aggregation process have been modelled and quantified. Visual and turbidity experiments showed that the thermal aggregation of rPA occurs at incubation temperatures ≥43°C, which is close to its denaturation temperature. Under these conditions, the protein is likely to have increased translational kinetic energy, hence more collisions will take place, and also be at least partially unfolded, hence, it has some exposed hydrophobic regions which are known to induce rapid and nonspecific aggregation. Such aggregation was found to proceed in a stepwise manner, by first forming spherical microscopic particles followed by clustering to form fractal aggregates. Increasing the temperature more than 43°C increased the rate of aggregation dramatically and also the size of the diameter of the spherical microscopic particles formed from ~360 to 500 nm when increasing the temperature from 43° to 49°C. We went on to show that the growth of the microscopic particles can be monitored using UV-LSS. In particular, we used the increase in scattered light from the sample over time to elucidate aggregate size versus time, giving a quantitative measure of the aggregation. Moreover, the experiments were conducted over a range of temperatures, with and without 0.25 M urea, and as such, the results were analysed to determine the rate constant and the temperature dependence of the thermal aggregation process. Based on this analysis, we proposed that the aggregation process is association limited and that the temperature dependence relates to the equilibrium behaviour between native and perturbed states. We were also able to extract the thermodynamic parameters for aggregation in samples with and without urea, and these indicated that the presence of urea reduces the stability of rPA, hence increases its susceptibility to aggregation. The modelling tools developed here for analysis of data from the easily accessible UV-LSS technique provides a fast between *in situ* analysis method for comparing the stability of different formulations of protein when exposed to different environmental conditions. This method has an important speed advantage over other light scattering techniques when analysing such particle growth kinetics. This work, therefore, provides a basis for quantitatively exploring the effect of additives and/or different processing conditions on the rate of aggregation of industrially relevant biopharmaceuticals with the aim of minimising any self-association during production, downstream processing, or storage. 

## Figures and Tables

**Table 1:** Refractive index of rPA particles at different wavelengths calculated using the Lorentz-Lorenz molar refraction.

Wavelength/nm	Refractive index, *n*
434.0	1.684
486.1	1.677
589.3	1.665
656.3	1.661

**Table 2:** Changes in enthalpy (Δ*H*), entropy (Δ*S*), Gibbs free energy (Δ*G*) at 25°C, and denaturation temperature (*T*_*m*_) for rPA unfolding (*N*↔*I*) with and without 0.25 M urea.

	Δ*H*/kJ mol^−1^	Δ*S*/kJ mol^−1^ K^−1^	Δ*G* at 25°C/kJ mol^−1^	*T*_*m*_ (Δ*G* = 0)/°C
rPA without additive	975.8 ± 40.5	3.02 ± 0.13	76.1	50.2
rPA with 0.25 M urea	949.7 ± 94.6	2.92 ± 0.30	77.7	51.6
